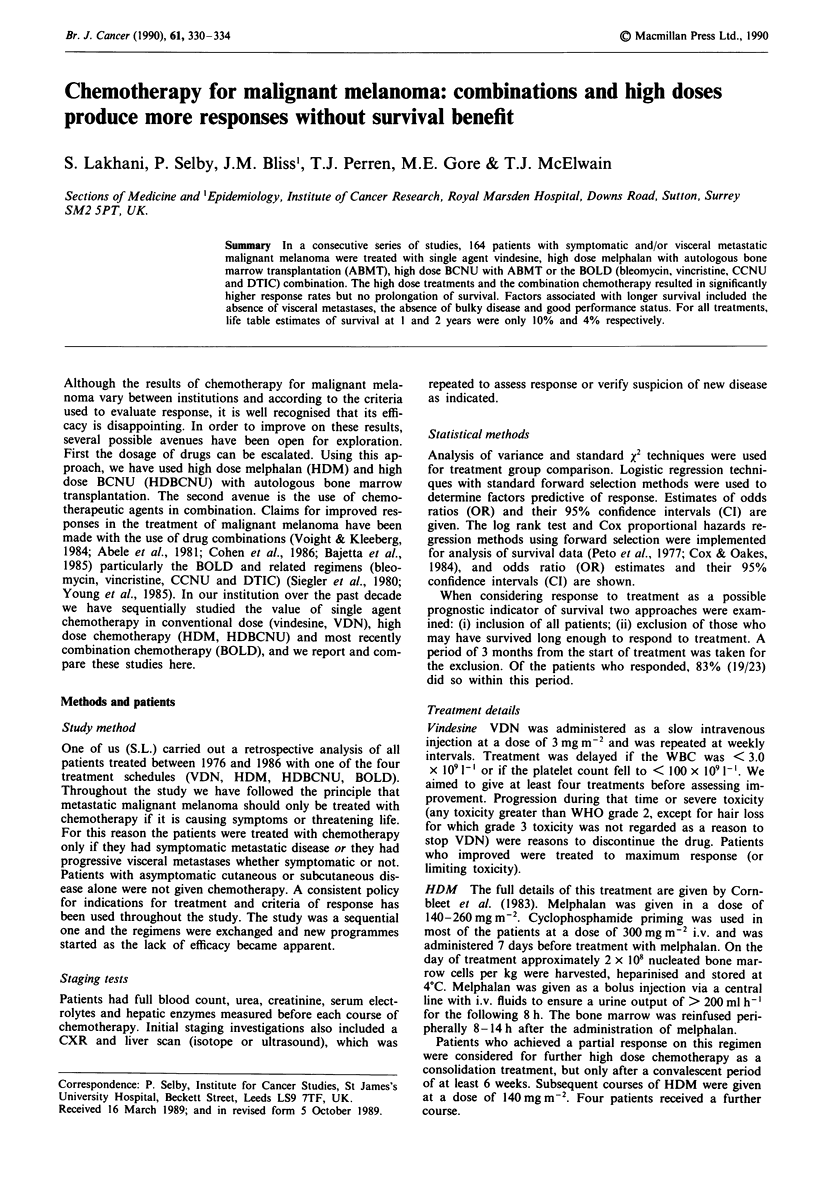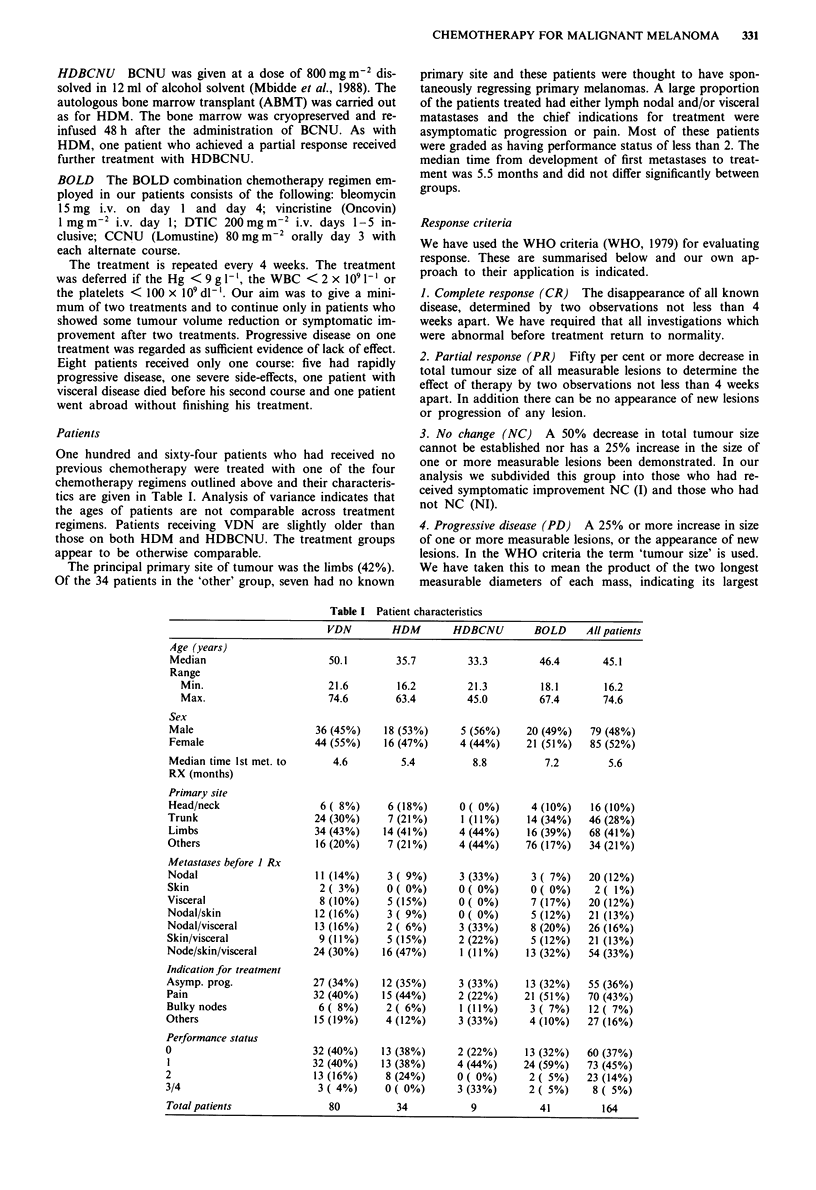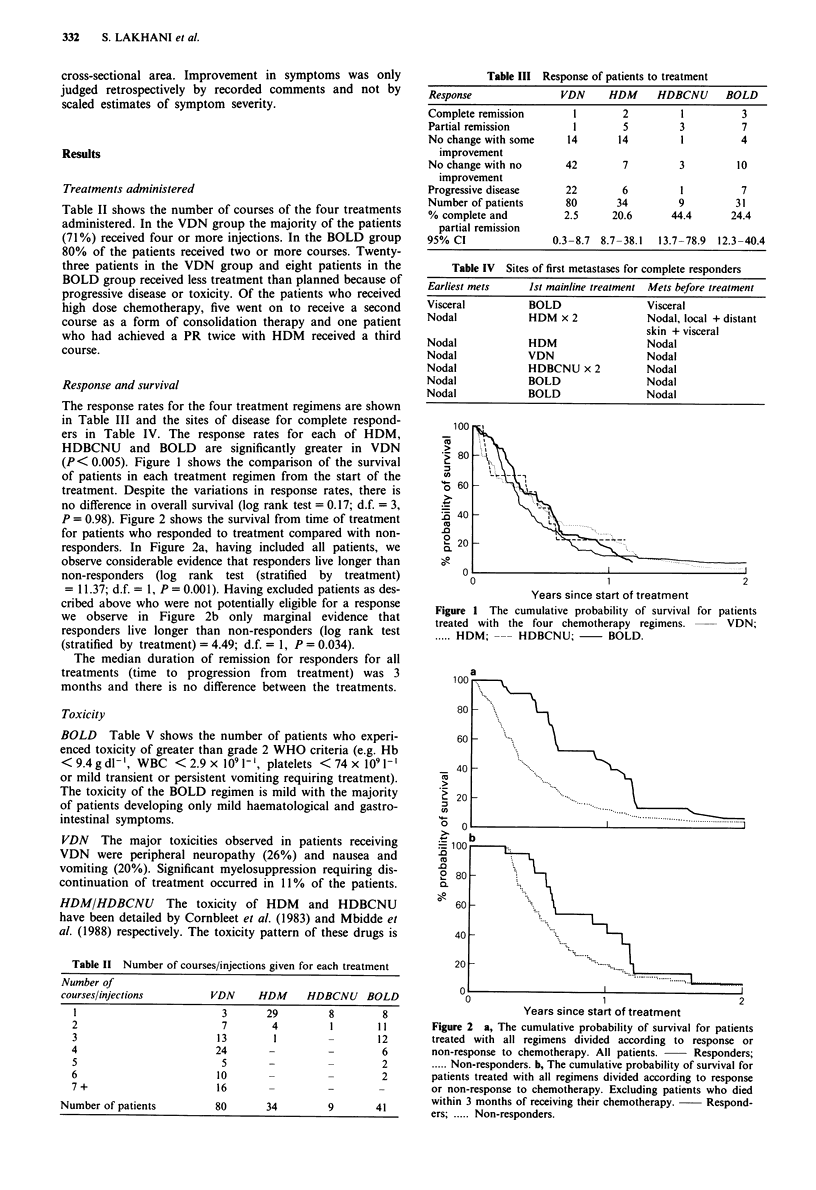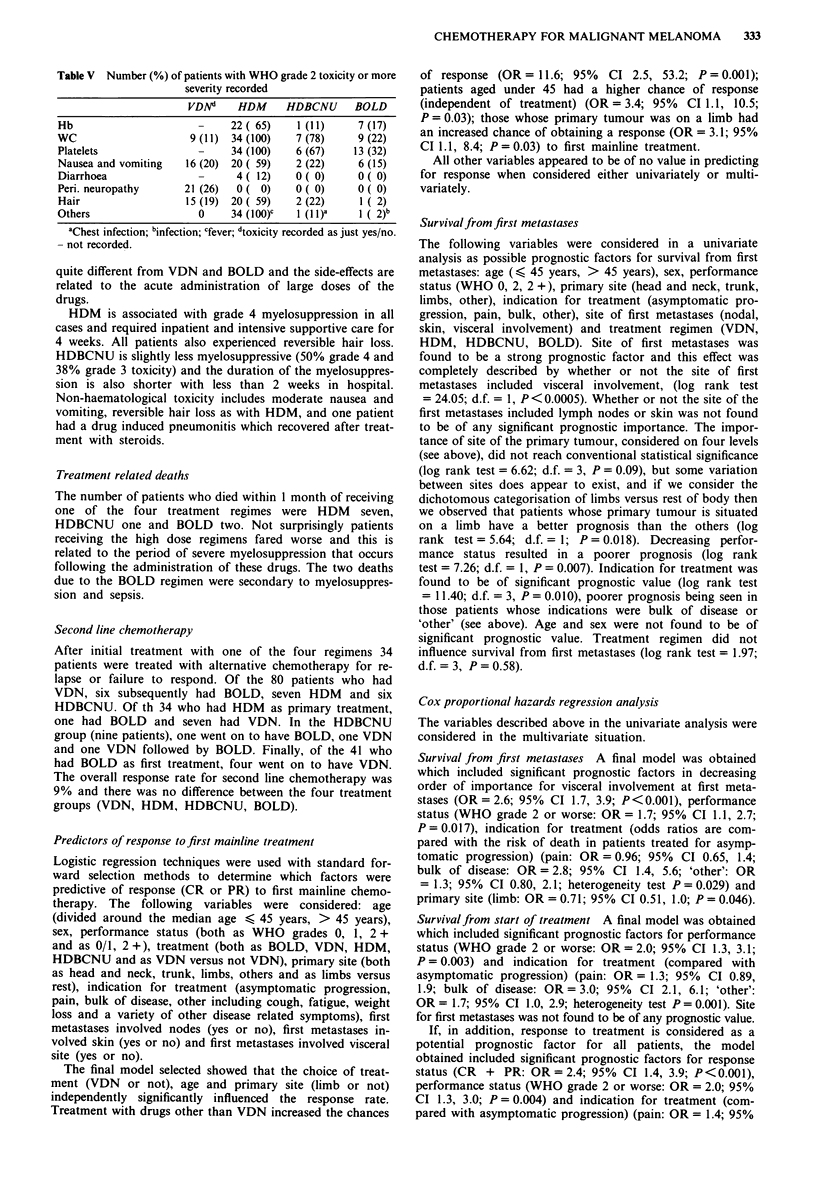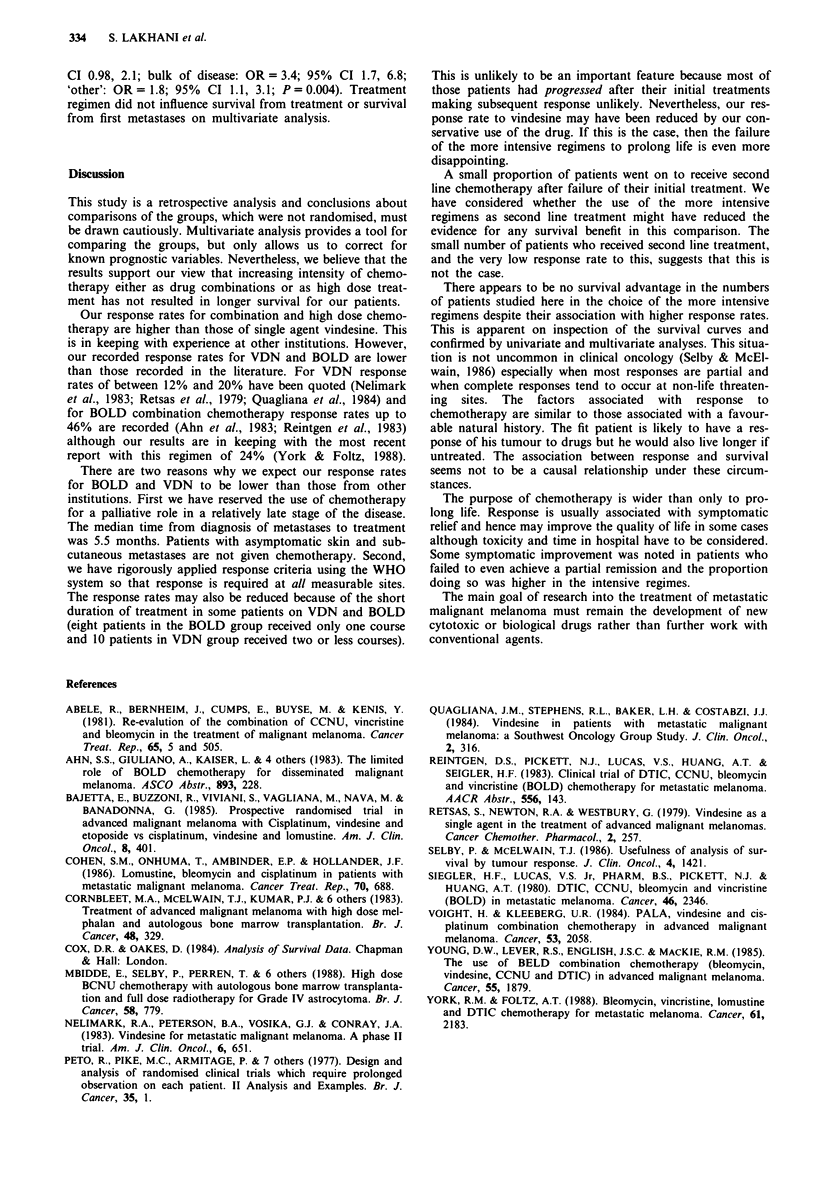# Chemotherapy for malignant melanoma: combinations and high doses produce more responses without survival benefit.

**DOI:** 10.1038/bjc.1990.65

**Published:** 1990-02

**Authors:** S. Lakhani, P. Selby, J. M. Bliss, T. J. Perren, M. E. Gore, T. J. McElwain

**Affiliations:** Section of Medicine, Royal Marsden Hospital, Sutton, Surrey, UK.

## Abstract

In a consecutive series of studies, 164 patients with symptomatic and/or visceral metastatic malignant melanoma were treated with single agent vindesine, high dose melphalan with autologous bone marrow transplantation (AMBT), high dose BCNU with ABMT or the BOLD (bleomycin, vincristine, CCNU and DTIC) combination. The high dose treatments and the combination chemotherapy resulted in significantly higher response rates but no prolongation of survival. Factors associated with longer survival included the absence of visceral metastases, the absence of bulky disease and good performance status. For all treatments, life table estimates of survival at 1 and 2 years were only 10% and 4% respectively.


Br. J. Cancer (1990), 61, 330-334 © Macmillan Press Ltd., 1990

### Chemotherapy for malignant melanoma: combinations and high doses produce more responses without survival benefit

S. Lakhani, P. Selby, J.M. Bliss[1], T.J. Perren, M.E. Gore & T.J. McElwain

*Sections of Medicine and [1]Epidemiology, Institute of Cancer Research, Royal Marsden Hospital, Downs Road, Sutton, Surrey SM2 5PT, UK.*

**Summary** In a consecutive series of studies, 164 patients with symptomatic and/or visceral metastatic malignant melanoma were treated with single agent vindesine, high dose melphalan with autologous bone marrow transplantation (ABMT), high dose BCNU with ABMT or the BOLD (bleomycin, vincristine, CCNU and DTIC) combination. The high dose treatments and the combination chemotherapy resulted in significantly higher response rates but no prolongation of survival. Factors associated with longer survival included the absence of visceral metastases, the absence of bulky disease and good performance status. For all treatments, life table estimates of survival at 1 and 2 years were only 10% and 4% respectively.

Although the results of chemotherapy for malignant melanoma vary between institutions and according to the criteria used to evaluate response, it is well recognised that its efficacy is disappointing. In order to improve on these results, several possible avenues have been open for exploration. First the dosage of drugs can be escalated. Using this approach, we have used high dose melphalan (HDM) and high dose BCNU (HDBCNU) with autologous bone marrow transplantation. The second avenue is the use of chemotherapeutic agents in combination. Claims for improved responses in the treatment of malignant melanoma have been made with the use of drug combinations (Voight & Kleeberg, 1984; Abele *et al.*, 1981; Cohen *et al.*, 1986; Bajetta *et al.*, 1985) particularly the BOLD and related regimens (bleomycin, vincristine, CCNU and DTIC) (Siegler *et al.*, 1980; Young *et al.*, 1985). In our institution over the past decade we have sequentially studied the value of single agent chemotherapy in conventional dose (vindesine, VDN), high dose chemotherapy (HDM, HDBCNU) and most recently combination chemotherapy (BOLD), and we report and compare these studies here.

### Methods and patients

### Study method

One of us (S.L.) carried out a retrospective analysis of all patients treated between 1976 and 1986 with one of the four treatment schedules (VDN, HDM, HDBCNU, BOLD). Throughout the study we have followed the principle that metastatic malignant melanoma should only be treated with chemotherapy if it is causing symptoms or threatening life. For this reason the patients were treated with chemotherapy only if they had symptomatic metastatic disease *or* they had progressive visceral metastases whether symptomatic or not. Patients with asymptomatic cutaneous or subcutaneous disease alone were not given chemotherapy. A consistent policy for indications for treatment and criteria of response has been used throughout the study. The study was a sequential one and the regimens were exchanged and new programmes started as the lack of efficacy became apparent.

### Staging tests

Patients had full blood count, urea, creatinine, serum electrolytes and hepatic enzymes measured before each course of chemotherapy. Initial staging investigations also included a CXR and liver scan (isotope or ultrasound), which was repeated to assess response or verify suspicion of new disease as indicated.

### Statistical methods

Analysis of variance and standard $\chi^2$ techniques were used for treatment group comparison. Logistic regression techniques with standard forward selection methods were used to determine factors predictive of response. Estimates of odds ratios (OR) and their 95% confidence intervals (CI) are given. The log rank test and Cox proportional hazards regression methods using forward selection were implemented for analysis of survival data (Peto *et al.*, 1977; Cox & Oakes, 1984), and odds ratio (OR) estimates and their 95% confidence intervals (CI) are shown.

When considering response to treatment as a possible prognostic indicator of survival two approaches were examined: (i) inclusion of all patients; (ii) exclusion of those who may have survived long enough to respond to treatment. A period of 3 months from the start of treatment was taken for the exclusion. Of the patients who responded, 83% (19/23) did so within this period.

### Treatment details

*Vindesine* VDN was administered as a slow intravenous injection at a dose of $3\ mg\ m^{-2}$ and was repeated at weekly intervals. Treatment was delayed if the WBC was $< 3.0 \times 10^9\ l^{-1}$ or if the platelet count fell to $< 100 \times 10^9\ l^{-1}$. We aimed to give at least four treatments before assessing improvement. Progression during that time or severe toxicity (any toxicity greater than WHO grade 2, except for hair loss for which grade 3 toxicity was not regarded as a reason to stop VDN) were reasons to discontinue the drug. Patients who improved were treated to maximum response (or limiting toxicity).

*HDM* The full details of this treatment are given by Cornbleet *et al.* (1983). Melphalan was given in a dose of $140-260\ mg\ m^{-2}$. Cyclophosphamide priming was used in most of the patients at a dose of $300\ mg\ m^{-2}$ i.v. and was administered 7 days before treatment with melphalan. On the day of treatment approximately $2 \times 10^8$ nucleated bone marrow cells per kg were harvested, heparinised and stored at 4°C. Melphalan was given as a bolus injection via a central line with i.v. fluids to ensure a urine output of $> 200\ ml\ h^{-1}$ for the following 8 h. The bone marrow was reinfused peripherally 8-14 h after the administration of melphalan.

Patients who achieved a partial response on this regimen were considered for further high dose chemotherapy as a consolidation treatment, but only after a convalescent period of at least 6 weeks. Subsequent courses of HDM were given at a dose of $140\ mg\ m^{-2}$. Four patients received a further course.

Correspondence: P. Selby, Institute for Cancer Studies, St James's University Hospital, Beckett Street, Leeds LS9 7TF, UK.
Received 16 March 1989; and in revised form 5 October 1989.

*HDBCNU* BCNU was given at a dose of 800 mg m$^{-2}$ dissolved in 12 ml of alcohol solvent (Mbidde *et al.*, 1988). The autologous bone marrow transplant (ABMT) was carried out as for HDM. The bone marrow was cryopreserved and reinfused 48 h after the administration of BCNU. As with HDM, one patient who achieved a partial response received further treatment with HDBCNU.

*BOLD* The BOLD combination chemotherapy regimen employed in our patients consists of the following: bleomycin 15 mg i.v. on day 1 and day 4; vincristine (Oncovin) 1 mg m$^{-2}$ i.v. day 1; DTIC 200 mg m$^{-2}$ i.v. days 1–5 inclusive; CCNU (Lomustine) 80 mg m$^{-2}$ orally day 3 with each alternate course.

The treatment is repeated every 4 weeks. The treatment was deferred if the Hg $< 9\ g\ l^{-1}$, the WBC $< 2 \times 10^9\ l^{-1}$ or the platelets $< 100 \times 10^9\ dl^{-1}$. Our aim was to give a minimum of two treatments and to continue only in patients who showed some tumour volume reduction or symptomatic improvement after two treatments. Progressive disease on one treatment was regarded as sufficient evidence of lack of effect. Eight patients received only one course: five had rapidly progressive disease, one severe side-effects, one patient with visceral disease died before his second course and one patient went abroad without finishing his treatment.

### *Patients*

One hundred and sixty-four patients who had received no previous chemotherapy were treated with one of the four chemotherapy regimens outlined above and their characteristics are given in Table I. Analysis of variance indicates that the ages of patients are not comparable across treatment regimens. Patients receiving VDN are slightly older than those on both HDM and HDBCNU. The treatment groups appear to be otherwise comparable.

The principal primary site of tumour was the limbs (42%). Of the 34 patients in the 'other' group, seven had no known primary site and these patients were thought to have spontaneously regressing primary melanomas. A large proportion of the patients treated had either lymph nodal and/or visceral matastases and the chief indications for treatment were asymptomatic progression or pain. Most of these patients were graded as having performance status of less than 2. The median time from development of first metastases to treatment was 5.5 months and did not differ significantly between groups.

### *Response criteria*

We have used the WHO criteria (WHO, 1979) for evaluating response. These are summarised below and our own approach to their application is indicated.

*1. Complete response (CR)* The disappearance of all known disease, determined by two observations not less than 4 weeks apart. We have required that all investigations which were abnormal before treatment return to normality.

*2. Partial response (PR)* Fifty per cent or more decrease in total tumour size of all measurable lesions to determine the effect of therapy by two observations not less than 4 weeks apart. In addition there can be no appearance of new lesions or progression of any lesion.

*3. No change (NC)* A 50% decrease in total tumour size cannot be established nor has a 25% increase in the size of one or more measurable lesions been demonstrated. In our analysis we subdivided this group into those who had received symptomatic improvement NC (I) and those who had not NC (NI).

*4. Progressive disease (PD)* A 25% or more increase in size of one or more measurable lesions, or the appearance of new lesions. In the WHO criteria the term 'tumour size' is used. We have taken this to mean the product of the two longest measurable diameters of each mass, indicating its largest

**Table I** Patient characteristics

| | *VDN* | *HDM* | *HDBCNU* | *BOLD* | *All patients* |
|---|---|---|---|---|---|
| *Age (years)* | | | | | |
| Median | 50.1 | 35.7 | 33.3 | 46.4 | 45.1 |
| Range | | | | | |
| Min. | 21.6 | 16.2 | 21.3 | 18.1 | 16.2 |
| Max. | 74.6 | 63.4 | 45.0 | 67.4 | 74.6 |
| *Sex* | | | | | |
| Male | 36 (45%) | 18 (53%) | 5 (56%) | 20 (49%) | 79 (48%) |
| Female | 44 (55%) | 16 (47%) | 4 (44%) | 21 (51%) | 85 (52%) |
| Median time 1st met. to RX (months) | 4.6 | 5.4 | 8.8 | 7.2 | 5.6 |
| *Primary site* | | | | | |
| Head/neck | 6 ( 8%) | 6 (18%) | 0 ( 0%) | 4 (10%) | 16 (10%) |
| Trunk | 24 (30%) | 7 (21%) | 1 (11%) | 14 (34%) | 46 (28%) |
| Limbs | 34 (43%) | 14 (41%) | 4 (44%) | 16 (39%) | 68 (41%) |
| Others | 16 (20%) | 7 (21%) | 4 (44%) | 76 (17%) | 34 (21%) |
| *Metastases before 1 Rx* | | | | | |
| Nodal | 11 (14%) | 3 ( 9%) | 3 (33%) | 3 ( 7%) | 20 (12%) |
| Skin | 2 ( 3%) | 0 ( 0%) | 0 ( 0%) | 0 ( 0%) | 2 ( 1%) |
| Visceral | 8 (10%) | 5 (15%) | 0 ( 0%) | 7 (17%) | 20 (12%) |
| Nodal/skin | 12 (16%) | 3 ( 9%) | 0 ( 0%) | 5 (12%) | 21 (13%) |
| Nodal/visceral | 13 (16%) | 2 ( 6%) | 3 (33%) | 8 (20%) | 26 (16%) |
| Skin/visceral | 9 (11%) | 5 (15%) | 2 (22%) | 5 (12%) | 21 (13%) |
| Node/skin/visceral | 24 (30%) | 16 (47%) | 1 (11%) | 13 (32%) | 54 (33%) |
| *Indication for treatment* | | | | | |
| Asymp. prog. | 27 (34%) | 12 (35%) | 3 (33%) | 13 (32%) | 55 (36%) |
| Pain | 32 (40%) | 15 (44%) | 2 (22%) | 21 (51%) | 70 (43%) |
| Bulky nodes | 6 ( 8%) | 2 ( 6%) | 1 (11%) | 3 ( 7%) | 12 ( 7%) |
| Others | 15 (19%) | 4 (12%) | 3 (33%) | 4 (10%) | 27 (16%) |
| *Performance status* | | | | | |
| 0 | 32 (40%) | 13 (38%) | 2 (22%) | 13 (32%) | 60 (37%) |
| 1 | 32 (40%) | 13 (38%) | 4 (44%) | 24 (59%) | 73 (45%) |
| 2 | 13 (16%) | 8 (24%) | 0 ( 0%) | 2 ( 5%) | 23 (14%) |
| 3/4 | 3 ( 4%) | 0 ( 0%) | 3 (33%) | 2 ( 5%) | 8 ( 5%) |
| *Total patients* | 80 | 34 | 9 | 41 | 164 |

cross-sectional area. Improvement in symptoms was only judged retrospectively by recorded comments and not by scaled estimates of symptom severity.

### Results

### *Treatments administered*

Table II shows the number of courses of the four treatments administered. In the VDN group the majority of the patients (71%) received four or more injections. In the BOLD group 80% of the patients received two or more courses. Twenty-three patients in the VDN group and eight patients in the BOLD group received less treatment than planned because of progressive disease or toxicity. Of the patients who received high dose chemotherapy, five went on to receive a second course as a form of consolidation therapy and one patient who had achieved a PR twice with HDM received a third course.

### *Response and survival*

The response rates for the four treatment regimens are shown in Table III and the sites of disease for complete responders in Table IV. The response rates for each of HDM, HDBCNU and BOLD are significantly greater in VDN ($P < 0.005$). Figure 1 shows the comparison of the survival of patients in each treatment regimen from the start of the treatment. Despite the variations in response rates, there is no difference in overall survival (log rank test = 0.17; d.f. = 3, $P = 0.98$). Figure 2 shows the survival from time of treatment for patients who responded to treatment compared with non-responders. In Figure 2a, having included all patients, we observe considerable evidence that responders live longer than non-responders (log rank test (stratified by treatment) = 11.37; d.f. = 1, $P = 0.001$). Having excluded patients as described above who were not potentially eligible for a response we observe in Figure 2b only marginal evidence that responders live longer than non-responders (log rank test (stratified by treatment) = 4.49; d.f. = 1, $P = 0.034$).

The median duration of remission for responders for all treatments (time to progression from treatment) was 3 months and there is no difference between the treatments.

### *Toxicity*

*BOLD* Table V shows the number of patients who experienced toxicity of greater than grade 2 WHO criteria (e.g. Hb $< 9.4\ g\ dl^{-1}$, WBC $< 2.9 \times 10^9\ l^{-1}$, platelets $< 74 \times 10^9\ l^{-1}$ or mild transient or persistent vomiting requiring treatment). The toxicity of the BOLD regimen is mild with the majority of patients developing only mild haematological and gastrointestinal symptoms.

*VDN* The major toxicities observed in patients receiving VDN were peripheral neuropathy (26%) and nausea and vomiting (20%). Significant myelosuppression requiring discontinuation of treatment occurred in 11% of the patients.

*HDM/HDBCNU* The toxicity of HDM and HDBCNU have been detailed by Cornbleet et al. (1983) and Mbidde et al. (1988) respectively. The toxicity pattern of these drugs is

**Table II** Number of courses/injections given for each treatment

| *Number of courses/injections* | *VDN* | *HDM* | *HDBCNU* | *BOLD* |
|---|---|---|---|---|
| 1 | 3 | 29 | 8 | 8 |
| 2 | 7 | 4 | 1 | 11 |
| 3 | 13 | 1 | – | 12 |
| 4 | 24 | – | – | 6 |
| 5 | 5 | – | – | 2 |
| 6 | 10 | – | – | 2 |
| 7+ | 16 | – | – | – |
| Number of patients | 80 | 34 | 9 | 41 |

**Table III** Response of patients to treatment

| *Response* | *VDN* | *HDM* | *HDBCNU* | *BOLD* |
|---|---|---|---|---|
| Complete remission | 1 | 2 | 1 | 3 |
| Partial remission | 1 | 5 | 3 | 7 |
| No change with some improvement | 14 | 14 | 1 | 4 |
| No change with no improvement | 42 | 7 | 3 | 10 |
| Progressive disease | 22 | 6 | 1 | 7 |
| Number of patients | 80 | 34 | 9 | 31 |
| % complete and partial remission | 2.5 | 20.6 | 44.4 | 24.4 |
| 95% CI | 0.3–8.7 | 8.7–38.1 | 13.7–78.9 | 12.3–40.4 |

**Table IV** Sites of first metastases for complete responders

| *Earliest mets* | *1st mainline treatment* | *Mets before treatment* |
|---|---|---|
| Visceral | BOLD | Visceral |
| Nodal | HDM x 2 | Nodal, local + distant skin + visceral |
| Nodal | HDM | Nodal |
| Nodal | VDN | Nodal |
| Nodal | HDBCNU x 2 | Nodal |
| Nodal | BOLD | Nodal |
| Nodal | BOLD | Nodal |

**Figure 1** The cumulative probability of survival for patients treated with the four chemotherapy regimens. —— VDN; ..... HDM; --- HDBCNU; —— BOLD.

**Figure 2** **a**, The cumulative probability of survival for patients treated with all regimens divided according to response or non-response to chemotherapy. All patients. —— Responders; ..... Non-responders. **b**, The cumulative probability of survival for patients treated with all regimens divided according to response or non-response to chemotherapy. Excluding patients who died within 3 months of receiving their chemotherapy. —— Responders; ..... Non-responders.

**Table V** Number (%) of patients with WHO grade 2 toxicity or more severity recorded

| | *VDN*[d] | *HDM* | *HDBCNU* | *BOLD* |
|---|---|---|---|---|
| Hb | – | 22 ( 65) | 1 (11) | 7 (17) |
| WC | 9 (11) | 34 (100) | 7 (78) | 9 (22) |
| Platelets | – | 34 (100) | 6 (67) | 13 (32) |
| Nausea and vomiting | 16 (20) | 20 ( 59) | 2 (22) | 6 (15) |
| Diarrhoea | – | 4 ( 12) | 0 ( 0) | 0 ( 0) |
| Peri. neuropathy | 21 (26) | 0 ( 0) | 0 ( 0) | 0 ( 0) |
| Hair | 15 (19) | 20 ( 59) | 2 (22) | 1 ( 2) |
| Others | 0 | 34 (100)[c] | 1 (11)[a] | 1 ( 2)[b] |

[a]Chest infection; [b]infection; [c]fever; [d]toxicity recorded as just yes/no. – not recorded.

quite different from VDN and BOLD and the side-effects are related to the acute administration of large doses of the drugs.

HDM is associated with grade 4 myelosuppression in all cases and required inpatient and intensive supportive care for 4 weeks. All patients also experienced reversible hair loss. HDBCNU is slightly less myelosuppressive (50% grade 4 and 38% grade 3 toxicity) and the duration of the myelosuppression is also shorter with less than 2 weeks in hospital. Non-haematological toxicity includes moderate nausea and vomiting, reversible hair loss as with HDM, and one patient had a drug induced pneumonitis which recovered after treatment with steroids.

*Treatment related deaths*

The number of patients who died within 1 month of receiving one of the four treatment regimes were HDM seven, HDBCNU one and BOLD two. Not surprisingly patients receiving the high dose regimens fared worse and this is related to the period of severe myelosuppression that occurs following the administration of these drugs. The two deaths due to the BOLD regimen were secondary to myelosuppression and sepsis.

*Second line chemotherapy*

After initial treatment with one of the four regimens 34 patients were treated with alternative chemotherapy for relapse or failure to respond. Of the 80 patients who had VDN, six subsequently had BOLD, seven HDM and six HDBCNU. Of th 34 who had HDM as primary treatment, one had BOLD and seven had VDN. In the HDBCNU group (nine patients), one went on to have BOLD, one VDN and one VDN followed by BOLD. Finally, of the 41 who had BOLD as first treatment, four went on to have VDN. The overall response rate for second line chemotherapy was 9% and there was no difference between the four treatment groups (VDN, HDM, HDBCNU, BOLD).

*Predictors of response to first mainline treatment*

Logistic regression techniques were used with standard forward selection methods to determine which factors were predictive of response (CR or PR) to first mainline chemotherapy. The following variables were considered: age (divided around the median age $\leqslant$ 45 years, $>$ 45 years), sex, performance status (both as WHO grades 0, 1, 2+ and as 0/1, 2+), treatment (both as BOLD, VDN, HDM, HDBCNU and as VDN versus not VDN), primary site (both as head and neck, trunk, limbs, others and as limbs versus rest), indication for treatment (asymptomatic progression, pain, bulk of disease, other including cough, fatigue, weight loss and a variety of other disease related symptoms), first metastases involved nodes (yes or no), first metastases involved skin (yes or no) and first metastases involved visceral site (yes or no).

The final model selected showed that the choice of treatment (VDN or not), age and primary site (limb or not) independently significantly influenced the response rate. Treatment with drugs other than VDN increased the chances of response (OR = 11.6; 95% CI 2.5, 53.2; $P = 0.001$); patients aged under 45 had a higher chance of response (independent of treatment) (OR = 3.4; 95% CI 1.1, 10.5; $P = 0.03$); those whose primary tumour was on a limb had an increased chance of obtaining a response (OR = 3.1; 95% CI 1.1, 8.4; $P = 0.03$) to first mainline treatment.

All other variables appeared to be of no value in predicting for response when considered either univariately or multivariately.

*Survival from first metastases*

The following variables were considered in a univariate analysis as possible prognostic factors for survival from first metastases: age ($\leqslant$ 45 years, $>$ 45 years), sex, performance status (WHO 0, 2, 2+), primary site (head and neck, trunk, limbs, other), indication for treatment (asymptomatic progression, pain, bulk, other), site of first metastases (nodal, skin, visceral involvement) and treatment regimen (VDN, HDM, HDBCNU, BOLD). Site of first metastases was found to be a strong prognostic factor and this effect was completely described by whether or not the site of first metastases included visceral involvement, (log rank test = 24.05; d.f. = 1, $P < 0.0005$). Whether or not the site of the first metastases included lymph nodes or skin was not found to be of any significant prognostic importance. The importance of site of the primary tumour, considered on four levels (see above), did not reach conventional statistical significance (log rank test = 6.62; d.f. = 3, $P = 0.09$), but some variation between sites does appear to exist, and if we consider the dichotomous categorisation of limbs versus rest of body then we observed that patients whose primary tumour is situated on a limb have a better prognosis than the others (log rank test = 5.64; d.f. = 1; $P = 0.018$). Decreasing performance status resulted in a poorer prognosis (log rank test = 7.26; d.f. = 1, $P = 0.007$). Indication for treatment was found to be of significant prognostic value (log rank test = 11.40; d.f. = 3, $P = 0.010$), poorer prognosis being seen in those patients whose indications were bulk of disease or 'other' (see above). Age and sex were not found to be of significant prognostic value. Treatment regimen did not influence survival from first metastases (log rank test = 1.97; d.f. = 3, $P = 0.58$).

*Cox proportional hazards regression analysis*

The variables described above in the univariate analysis were considered in the multivariate situation.

*Survival from first metastases* A final model was obtained which included significant prognostic factors in decreasing order of importance for visceral involvement at first metastases (OR = 2.6; 95% CI 1.7, 3.9; $P < 0.001$), performance status (WHO grade 2 or worse: OR = 1.7; 95% CI 1.1, 2.7; $P = 0.017$), indication for treatment (odds ratios are compared with the risk of death in patients treated for asymptomatic progression) (pain: OR = 0.96; 95% CI 0.65, 1.4; bulk of disease: OR = 2.8; 95% CI 1.4, 5.6; 'other': OR = 1.3; 95% CI 0.80, 2.1; heterogeneity test $P = 0.029$) and primary site (limb: OR = 0.71; 95% CI 0.51, 1.0; $P = 0.046$).

*Survival from start of treatment* A final model was obtained which included significant prognostic factors for performance status (WHO grade 2 or worse: OR = 2.0; 95% CI 1.3, 3.1; $P = 0.003$) and indication for treatment (compared with asymptomatic progression) (pain: OR = 1.3; 95% CI 0.89, 1.9; bulk of disease: OR = 3.0; 95% CI 2.1, 6.1; 'other': OR = 1.7; 95% CI 1.0, 2.9; heterogeneity test $P = 0.001$). Site for first metastases was not found to be of any prognostic value.

If, in addition, response to treatment is considered as a potential prognostic factor for all patients, the model obtained included significant prognostic factors for response status (CR + PR: OR = 2.4; 95% CI 1.4, 3.9; $P < 0.001$), performance status (WHO grade 2 or worse: OR = 2.0; 95% CI 1.3, 3.0; $P = 0.004$) and indication for treatment (compared with asymptomatic progression) (pain: OR = 1.4; 95%

CI 0.98, 2.1; bulk of disease: OR = 3.4; 95% CI 1.7, 6.8; 'other': OR = 1.8; 95% CI 1.1, 3.1; $P = 0.004$). Treatment regimen did not influence survival from treatment or survival from first metastases on multivariate analysis.

### Discussion

This study is a retrospective analysis and conclusions about comparisons of the groups, which were not randomised, must be drawn cautiously. Multivariate analysis provides a tool for comparing the groups, but only allows us to correct for known prognostic variables. Nevertheless, we believe that the results support our view that increasing intensity of chemotherapy either as drug combinations or as high dose treatment has not resulted in longer survival for our patients.

Our response rates for combination and high dose chemotherapy are higher than those of single agent vindesine. This is in keeping with experience at other institutions. However, our recorded response rates for VDN and BOLD are lower than those recorded in the literature. For VDN response rates of between 12% and 20% have been quoted (Nelimark *et al.*, 1983; Retsas *et al.*, 1979; Quagliana *et al.*, 1984) and for BOLD combination chemotherapy response rates up to 46% are recorded (Ahn *et al.*, 1983; Reintgen *et al.*, 1983) although our results are in keeping with the most recent report with this regimen of 24% (York & Foltz, 1988).

There are two reasons why we expect our response rates for BOLD and VDN to be lower than those from other institutions. First we have reserved the use of chemotherapy for a palliative role in a relatively late stage of the disease. The median time from diagnosis of metastases to treatment was 5.5 months. Patients with asymptomatic skin and subcutaneous metastases are not given chemotherapy. Second, we have rigorously applied response criteria using the WHO system so that response is required at *all* measurable sites. The response rates may also be reduced because of the short duration of treatment in some patients on VDN and BOLD (eight patients in the BOLD group received only one course and 10 patients in VDN group received two or less courses). This is unlikely to be an important feature because most of those patients had *progressed* after their initial treatments making subsequent response unlikely. Nevertheless, our response rate to vindesine may have been reduced by our conservative use of the drug. If this is the case, then the failure of the more intensive regimens to prolong life is even more disappointing.

A small proportion of patients went on to receive second line chemotherapy after failure of their initial treatment. We have considered whether the use of the more intensive regimens as second line treatment might have reduced the evidence for any survival benefit in this comparison. The small number of patients who received second line treatment, and the very low response rate to this, suggests that this is not the case.

There appears to be no survival advantage in the numbers of patients studied here in the choice of the more intensive regimens despite their association with higher response rates. This is apparent on inspection of the survival curves and confirmed by univariate and multivariate analyses. This situation is not uncommon in clinical oncology (Selby & McElwain, 1986) especially when most responses are partial and when complete responses tend to occur at non-life threatening sites. The factors associated with response to chemotherapy are similar to those associated with a favourable natural history. The fit patient is likely to have a response of his tumour to drugs but he would also live longer if untreated. The association between response and survival seems not to be a causal relationship under these circumstances.

The purpose of chemotherapy is wider than only to prolong life. Response is usually associated with symptomatic relief and hence may improve the quality of life in some cases although toxicity and time in hospital have to be considered. Some symptomatic improvement was noted in patients who failed to even achieve a partial remission and the proportion doing so was higher in the intensive regimes.

The main goal of research into the treatment of metastatic malignant melanoma must remain the development of new cytotoxic or biological drugs rather than further work with conventional agents.

### References

ABELE, R., BERNHEIM, J., CUMPS, E., BUYSE, M. & KENIS, Y. (1981). Re-evalution of the combination of CCNU, vincristine and bleomycin in the treatment of malignant melanoma. *Cancer Treat. Rep.*, **65**, 5 and 505.

AHN, S.S., GIULIANO, A., KAISER, L. & 4 others (1983). The limited role of BOLD chemotherapy for disseminated malignant melanoma. *ASCO Abstr.*, **893**, 228.

BAJETTA, E., BUZZONI, R., VIVIANI, S., VAGLIANA, M., NAVA, M. & BANADONNA, G. (1985). Prospective randomised trial in advanced malignant melanoma with Cisplatinum, vindesine and etoposide vs cisplatinum, vindesine and lomustine. *Am. J. Clin. Oncol.*, **8**, 401.

COHEN, S.M., ONHUMA, T., AMBINDER, E.P. & HOLLANDER, J.F. (1986). Lomustine, bleomycin and cisplatinum in patients with metastatic malignant melanoma. *Cancer Treat. Rep.*, **70**, 688.

CORNBLEET, M.A., MCELWAIN, T.J., KUMAR, P.J. & 6 others (1983). Treatment of advanced malignant melanoma with high dose melphalan and autologous bone marrow transplantation. *Br. J. Cancer*, **48**, 329.

COX, D.R. & OAKES, D. (1984). *Analysis of Survival Data*. Chapman & Hall: London.

MBIDDE, E., SELBY, P., PERREN, T. & 6 others (1988). High dose BCNU chemotherapy with autologous bone marrow transplantation and full dose radiotherapy for Grade IV astrocytoma. *Br. J. Cancer*, **58**, 779.

NELIMARK, R.A., PETERSON, B.A., VOSIKA, G.J. & CONRAY, J.A. (1983). Vindesine for metastatic malignant melanoma. A phase II trial. *Am. J. Clin. Oncol.*, **6**, 651.

PETO, R., PIKE, M.C., ARMITAGE, P. & 7 others (1977). Design and analysis of randomised clinical trials which require prolonged observation on each patient. II Analysis and Examples. *Br. J. Cancer*, **35**, 1.

QUAGLIANA, J.M., STEPHENS, R.L., BAKER, L.H. & COSTABZI, J.J. (1984). Vindesine in patients with metastatic malignant melanoma: a Southwest Oncology Group Study. *J. Clin. Oncol.*, **2**, 316.

REINTGEN, D.S., PICKETT, N.J., LUCAS, V.S., HUANG, A.T. & SEIGLER, H.F. (1983). Clinical trial of DTIC, CCNU, bleomycin and vincristine (BOLD) chemotherapy for metastatic melanoma. *AACR Abstr.*, **556**, 143.

RETSAS, S., NEWTON, R.A. & WESTBURY, G. (1979). Vindesine as a single agent in the treatment of advanced malignant melanomas. *Cancer Chemother. Pharmacol.*, **2**, 257.

SELBY, P. & MCELWAIN, T.J. (1986). Usefulness of analysis of survival by tumour response. *J. Clin. Oncol.*, **4**, 1421.

SIEGLER, H.F., LUCAS, V.S. Jr, PHARM, B.S., PICKETT, N.J. & HUANG, A.T. (1980). DTIC, CCNU, bleomycin and vincristine (BOLD) in metastatic melanoma. *Cancer*, **46**, 2346.

VOIGHT, H. & KLEEBERG, U.R. (1984). PALA, vindesine and cisplatinum combination chemotherapy in advanced malignant melanoma. *Cancer*, **53**, 2058.

YOUNG, D.W., LEVER, R.S., ENGLISH, J.S.C. & MACKIE, R.M. (1985). The use of BELD combination chemotherapy (bleomycin, vindesine, CCNU and DTIC) in advanced malignant melanoma. *Cancer*, **55**, 1879.

YORK, R.M. & FOLTZ, A.T. (1988). Bleomycin, vincristine, lomustine and DTIC chemotherapy for metastatic melanoma. *Cancer*, **61**, 2183.